# Influence of chemical disorder on energy dissipation and defect evolution in concentrated solid solution alloys

**DOI:** 10.1038/ncomms9736

**Published:** 2015-10-28

**Authors:** Yanwen Zhang, G. Malcolm Stocks, Ke Jin, Chenyang Lu, Hongbin Bei, Brian C. Sales, Lumin Wang, Laurent K. Béland, Roger E. Stoller, German D. Samolyuk, Magdalena Caro, Alfredo Caro, William J. Weber

**Affiliations:** 1 Materials Science and Technology Division, Oak Ridge National Laboratory, Oak Ridge, Tennessee 37831, USA; 2 Department of Materials Science and Engineering, University of Tennessee, Knoxville, Tennessee 37996, USA; 3 Department of Nuclear Engineering and Radiological Sciences, University of Michigan, Ann Arbor, Michigan 48109-2104, USA; 4 Los Alamos National Laboratory, Los Alamos, New Mexico 87545, USA

## Abstract

A grand challenge in materials research is to understand complex electronic correlation and non-equilibrium atomic interactions, and how such intrinsic properties and dynamic processes affect energy transfer and defect evolution in irradiated materials. Here we report that chemical disorder, with an increasing number of principal elements and/or altered concentrations of specific elements, in single-phase concentrated solid solution alloys can lead to substantial reduction in electron mean free path and orders of magnitude decrease in electrical and thermal conductivity. The subsequently slow energy dissipation affects defect dynamics at the early stages, and consequentially may result in less deleterious defects. Suppressed damage accumulation with increasing chemical disorder from pure nickel to binary and to more complex quaternary solid solutions is observed. Understanding and controlling energy dissipation and defect dynamics by altering alloy complexity may pave the way for new design principles of radiation-tolerant structural alloys for energy applications.

## Abstract (web summary)

The understanding of complex electronic correlation and non-equilibrium atomic interactions is a grand challenge. Here, the authors show that chemical disorder in single-phase concentrated solid solution alloys can lead to reduction in electron mean free path and electrical and thermal conductivity.

## Graphical abstract

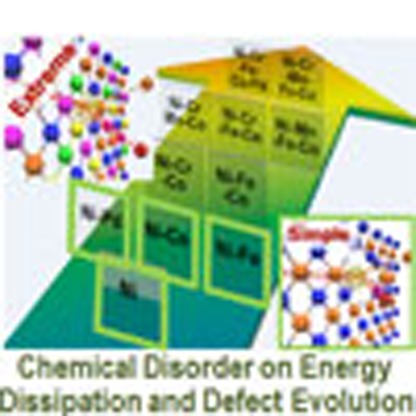

The development of metallic alloys is arguably one of the oldest sciences, dating back at least 3,000 years. Most research and applications have been focused on alloys with one principal element, to which the addition of alloying elements in low concentrations leads to various performance improvements and changes in radiation resistance (Supplementary Note 1). A quarter of a century-old observation has shown that fewer voids form in pure nickel under ion irradiation with Cu ions than with Ni ions, indicating possible chemical effects of substituting Cu into nickel lattice^1^,^2^. In the Ni–Al binary alloy system^3^, irradiation-induced microstructural and density changes were found to depend heavily on both Al content and irradiation temperature, suggesting significant modification of defect processes. Although the importance of Al content is clearly demonstrated, the underlying mechanisms of energy dissipation and defect dynamics are not known. The Ni–Cr systems with low concentration of other alloying elements or impurities were developed for nuclear energy applications^4^,^5^,^6^; irradiation studies indicate a low quasi-stationary concentration of primary point defects, which may be attributed to either reduced defect production or enhanced recombination that can be an intrinsic feature associated with the alloy composition and local chemistry. The size effect of solute atoms (Si, Co, Cu, Mn and Pd) in nickel^7^ has been investigated under He irradiation, and the results suggest that both the irradiation-induced point defects and the mobility of He are influenced by solute atoms, structural distortion and chemical disorder. The above brief overview of microstructure changes in various alloys reveals the consequences of complex interactions at the level of electrons and atoms in complicated alloys (in many cases containing additional phases, interfaces, grain boundaries, nanoparticles, etc.) over a relatively long evolution. More fundamentally, the alloy development and progress represent a major scientific challenge: can we take advantage of property enhancements to go beyond linear extrapolation, to develop a fundamental understanding of energy dissipation mechanisms and to control defect evolution? The long-standing challenge and tangled outcomes from alloy development guide us to focus our work on the early stages of defect dynamics in simple lattice structure with an overarching goal to understand the correlation between intrinsic properties, energy dissipation and defect evolution.

Recent success in the synthesis of single-phase concentrated solid solution alloys (SP-CSAs) has opened up new frontiers in materials research^8^,^9^,^10^,^11^,^12^,^13^,^14^,^15^,^16^,^17^,^18^,^19^. These alloys possess exceptional mechanical properties compared with conventional alloys, including high-temperature thermal stability and hardness, improved fatigue and fracture resistance, excellent corrosion and wear resistance, and high strength-to-weight ratio. The SP-CSAs, in contrast to traditional alloy systems, are random solid solutions based on simple underlying face-centred cubic (fcc) or body-centred cubic (bcc) crystal structures with two or more multiple principal elements. SP-CSAs with five or more components at equiatomic ratios are also referred to as high-entropy alloys (HEAs). SP-CSAs stand in sharp contrast to traditional binary and ternary alloy systems normally containing multiple phases with complex microstructures, of which numerous nickel- and iron-based alloys are classic examples. Random arrangement of multiple elemental species in an ordered structure (fcc or bcc), as in SP-CSAs, results in local disordered chemical environments and unique site-to-site lattice distortions. As a result of this site occupancy chemical disorder, SP-CSAs can also possess extreme complexity at the electron level. Moreover, heat conduction in SP-CSAs via electrons and lattice vibration should be effectively reduced due to this intrinsic chemical disorder, and the production and lifetimes of defects and their agglomeration under irradiation are expected to be different from those processes in traditional alloys. Lower defect production and higher annealing rates, along with desired intrinsic properties, may make it possible to yield systems with an inherent tendency for self-recovery.

Despite the recent success in alloy synthesis^8^,^9^,^10^,^11^,^12^,^13^,^14^,^15^,^16^,^17^, the scientific understanding of strongly disordered SP-CSAs, especially the defect physics, lags far behind the technical exploration. The use of ion beams to synthesize and functionalize materials or study the response of materials to extreme radiation environments has evolved over the past several decades^20^,^21^,^22^,^23^,^24^. In a radiation environment, the interaction of energetic ions with solids results in energy deposition to both electrons and atomic nuclei. Chemical disorder and compositional fluctuations induced by extreme alloy complexity can have significant impact on energy dissipation and defect evolution, and therefore radiation resistance. As SP-CSAs possess unique links between intrinsic material properties, energy dissipation and various dynamic processes, they are ideal systems to fill the knowledge gaps between electronic-/atomic-level interactions and radiation resistance mechanisms. Contrary to conventional alloys with low impurity concentration but multiple phases, controlling energy dissipation at the level of electrons and atoms in SP-CSA systems with extreme compositional disorder is unexplored territory.

In this work, we show that chemical disorder and compositional complexity in SP-CSAs have an enormous impact on defect dynamics through the substantial modification of energy dissipation pathways. Four model fcc single crystals of elemental nickel, binaries NiFe and NiCo, and quaternary NiCoFeCr, all at equiatomic ratios, are chosen for investigation. By performing *ab initio* electronic structure calculations, predicting primary damage formation in displacement cascades and lattice thermal conductivity based on molecular dynamics simulations, understanding intrinsic transport properties based on electrical resistivity and total thermal conductivity measurements, and identifying irradiation response utilizing *in situ* ion channelling technique and high-resolution transmission electron microscopy (TEM), we show that increasing chemical disorder in these model alloys can lead to substantial reduction in the electron mean free path and thermal conductivity. Furthermore, that much slower energy dissipation results in less deleterious defects and consequent slower damage accumulation under ion irradiation.

## Results

### Electronic structure calculations

In these model single crystals, the existence of elemental variation leads to intrinsic chemical disorder and atomic position distortion. Because every atom has a different local environment, no single atom actually resides on an ideal lattice site. With reference to the perfect fcc lattice structure, these small displacements can be viewed as displacement fluctuations or lattice distortions. From the point of view their electronic structure, the highly concentrated solid solution alloys of interest in this work are fundamentally different form pure metals or ordered compounds. The underlying fcc crystal structure in these SP-CSAs is only well defined on average, and their properties are dominated by their intrinsic chemical disorder and the local lattice distortions that results from the fact that every atom is surrounded by its own distinct chemical environment. Even in thermal equilibrium, the intrinsic potential scattering of different atomic species and the additional scattering due to positional or displacement fluctuations away from the ideal fcc lattice have important impacts on all electronic, atomic, and magnetic properties. Most important, the lack of translational symmetry in multi-component SP-CSAs that results from random site occupancy by different elements means Bloch states are no longer eigenstates of the system; the electronic band structure of the ordered system becomes smeared out in reciprocal (***k***) space.

**Figure 1:**
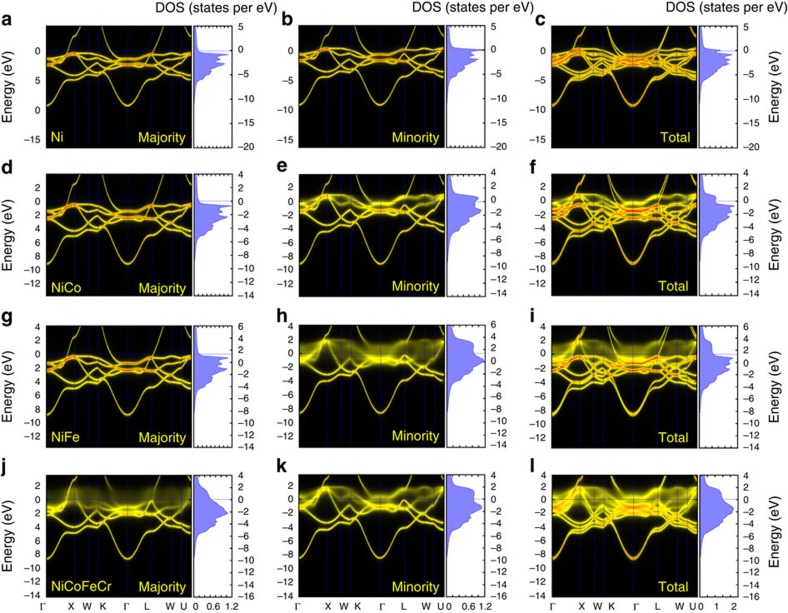
Electronic structure calculated using KKR-CPA method. Bloch spectral density function along high-symmetry directions and density of states of (**a**–**c**) nickel, (**d**–**f**) NiCo, (**g**–**i**) NiFe and (**j**–**l**) NiCoFeCr. Each of the 12 plots shows the disorder broadening of the electronic structure as encapsulated in the Bloch spectral function (left of panels) and the corresponding densities of states (right of panels). **a**,**d**,**g**,**j** and **b**,**e**,**h**,**k** show the majority (spin up) and minority (spin down) BSF and DOS, while **c**,**f**,**i**,**l** show the corresponding total BSF and DOS. The DOS is plotted in units of states per eV per atom.

To characterize the effects of chemical disorder on the electronic and magnetic properties, we performed electron structure calculations using *ab initio* Korringa–Kohn–Rostoker coherent-potential-approximation (KKR-CPA) method. The method, implemented within density functional theory, provides an *ab initio* theoretical description of the effects of disorder on the underlying electronic structure^25^. An important aspect of the KKR-CPA is that it is specifically formulated to calculate the configurationally averaged electronic structure, including local and total densities of states (DOS) and magnetic moments within a single-site (or mean-field) theory. Moreover, the KKR-CPA has also been extended to calculate many other properties, including electron transport^26^,^27^,^28^. Here it is worth noting that it is the configurationally averaged properties of random solid solutions that are actually measured by experiments^25^. Figure 1 shows the Bloch spectral function (BSF), which is a generalization of the band structure of an ordered system to include disorder, and configurationally averaged DOS for nickel, NiCo, NiFe and NiCoFeCr. In Fig. 1, the right-hand panels show the total BSF and DOS for each alloy, while the left-hand and centre panels show the contributions from the spin-up (majority) and spin-down (minority) electrons, respectively. As can be seen from the BSF plots, whereas nickel has well-defined bands as expected for pure metals, the band structures of the alloys are smeared out near the Fermi energy. For NiCo and NiFe the d-band smearing is largely limited to minority states; whereas in NiCoFeCr, both minority and majority states are smeared, giving rise to the much larger overall smearing (Fig. 1 right column). The Fermi energy wave vector broadening of the BSF is related to the inverse of the electron mean free path. At the Fermi energy, k-space smearing implies a decrease in electron mean free path. No (or little) smearing implies an infinite (long) mean free path, whereas uniform smearing throughout the Brillouin zone implies a mean free path on the order of the interatomic spacing. While the electron mean free path for the binary alloys (Fig. 1b,c) is short for the minority spin electrons, it is large for the majority spin electrons thereby providing a short circuit and an overall low resistivity. On the contrary, for the four-element NiCoFeCr alloy (Fig. 1d), both channels are broadened, particularly near the Fermi energy, implying a short mean free path in both spin channels. The consequently strong electron scattering can lead to a high residual resistivity.

It is important to note that in our KKR-CPA calculations we assume that the atoms are situated (on average) on an ideal fcc lattice. Consequently, the disorder smearing seen in the BSF results entirely from species and spin-dependent potential scattering. Indeed, fully relaxed 256-atom super-cell models of the disordered alloy, in which the atomic species are initially randomly distributed on an ideal fcc lattice, reveal that the displacement fluctuations are rather small: a maximum of ∼6%, average of ∼1.5%, of the nearest-neighbour distance, even in NiCoFeCr. The results shown in Fig. 1 suggest that large degrees of disorder smearing, and much shorter mean free paths than in elemental nickel, can already occur in the binary and quaternary alloys due to onsite chemical disorder alone. As a result, significant reduction of the effectiveness of energy dissipation through the electronic subsystem is expected.

### Transport property measurements and predictions

**Figure 2:**
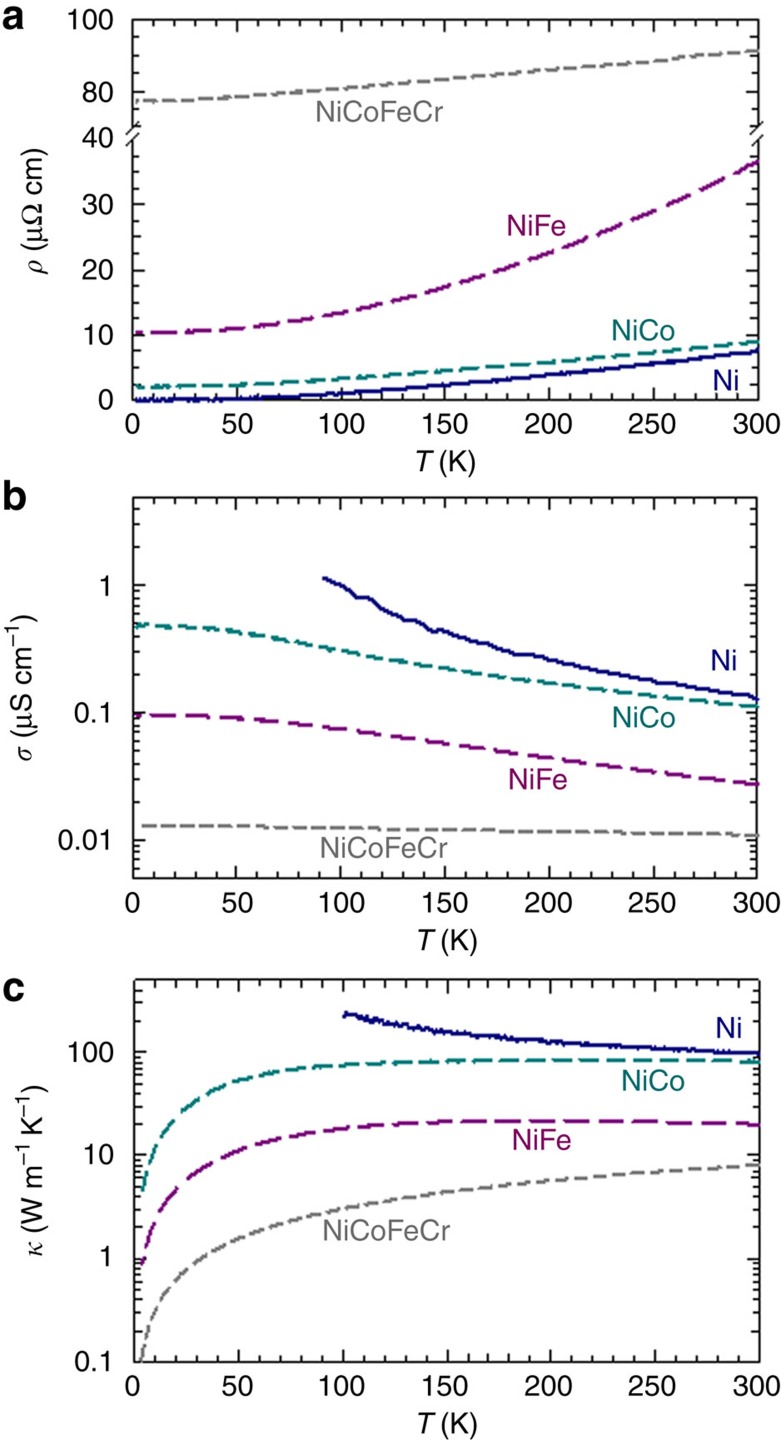
Electronic transport properties. Electrical resistivity (**a**), electrical conductivity (**b**) and electronic thermal conductivity estimated from the Wiedemann-Franz law (**c**). **b** and **c** are plotted in logarithmic scale for clarity.

Heat conduction in metals and alloys depends on how far the valence electrons can travel before they are scattered and change direction, and is closely related to electrical conductivity. The intrinsic electrical resistivity at zero temperature (residual resistivity) reflects the extent of intrinsic disorder scattering (broadening of the Fermi energy BSF) and at finite temperatures includes an additional contribution from electron–phonon scattering. The results of electrical resistivity measurements between 5 K and room temperature are shown in Fig. 2a, and the corresponding electrical conductivity is shown in Fig. 2b. Orders of magnitude increase in temperature-dependent resistivity are observed between nickel and the quaternary alloys. At 5 K, the resistivity ranges from∼0.1 μΩ cm for nickel to 2.0, 10.3 and 77.1 μΩ cm for NiCo, NiFe and NiCoFeCr, respectively. At the intermediate temperature of 100 K, there is an increase in resistivity and the corresponding values are 0.97, 3.35, 13.4 and 80.6 μΩ cm. When temperature increases to 300 K, the resistivity reaches 7.8, 9.0, 36.5 and 91.1 μΩ cm for nickel, NiCo, NiFe and NiCoFeCr, respectively. Clearly, the large changes in the residual resistivity values of NiCo, NiFe and NiCoFeCr are consistent with the increasing BSF smearing seen in Fig. 1.

Total thermal conductivity has both electronic and lattice (phonon) contributions. For pure metals, the heat is carried mainly by the valence electrons, and the conduction by phonons is significantly smaller than that by electrons at all temperatures^29^. In alloys, either electrons or phonons may be the principal carriers of thermal energy. In traditional alloys, the electrons are scattered mainly by the solute elements at low temperatures. Lattice thermal conductivity is often comparable to and sometimes even greater than the electronic component at low temperatures, and is not negligible even at temperatures well above the Debye temperature, 470 K for iron and 450 K for nickel, respectively^30^. In strongly disordered systems, however, the electron mean free path can be much reduced and thus we can expect a consequent reduction of electrical thermal conductivity in SP-CSAs, as shown in Fig. 1.

The electrical thermal conductivity (Fig. 2c) of the model crystals is estimated from their measured resistivity (Fig. 2a) or electrical conductivity (Fig. 2b) using the Wiedemann–Franz law. For pure nickel, the mean free path is long, so the electrical conductivity and thermal conductivity are high at low temperatures. With increasing temperature, the mean free path decreases resulting from increasing lattice vibration, therefore both the electrical conductivity and the electrical thermal conductivity tend to decrease with temperature. In the SP-CSAs, the reduced electrical thermal conductivity, compared with that of nickel, is attributed to the band structure effects and the enhanced disorder scattering (Fig. 1). A difference in temperature dependence is also observed in SP-CSAs. Whereas the electrical thermal conductivity of both binaries increases at low temperatures and saturates when the temperature approaches room temperature, it increases linearly in the quaternary alloy. Moreover, the impact of specific alloying elements on electrical conductivity and thermal conductivity is clearly indicated by the much larger values of NiCo (∼4 times) than NiFe with increasing temperatures. The spin-polarized KKR-CPA calculations provide information on magnetic ground states. As discussed earlier, the significant increase in resistivity between the two binaries and the four-component alloy is due to the broadening of both majority and minority channels in NiCoFeCr alloy (no short circuit). In the binaries (Fig. 1b,c), while the DOS and BSF both exhibit sharp structure for the majority spin channel, very similar to pure nickel (Fig. 1a), the DOS are broadened in energy and wave vector for the minority spin channel. As the d-states in NiFe are significantly broader in comparison to NiCo in the BSF plots, much stronger electron scattering (shorter electron mean free path) in NiFe is therefore expected. This prediction is in line with the electrical conductivity and electrical thermal conductivity shown in Fig. 2b,c. The results on pure Ni and the binaries shown in Figs 1 and 2 suggest that while alloy complexity can result from the increasing number of principal elements, the level of chemical disorder may depend substantively on the specific alloying elements.

To estimate the relative roles of potential scattering from chemical disorder and from displacement fluctuations in SP-CSAs, the lattice thermal conductivity of nickel, NiFe and NiFeCrCo was estimated via molecular dynamics simulations using the Green–Kubo formalism^31^ and classical potentials^32^. The temperature-dependent lattice thermal conductivity is normally described in three temperature regions: below, about and above the temperature at which the conductivity has its maximum value; this temperature depends on the alloy composition^29^. At 300 K the electrical thermal conductivities of nickel, NiCo, NiFe and NiCoFeCr obtained from the measured electrical conductivities and the Wiedemann–Franz relation are ∼88, 74, 21.1 and 8.0 W m^−1^ K^−1^, respectively (Fig. 2c). The total thermal conductivity of pure nickel is reported to be 90.9 W m^−1^ K^−1^ (ref. 33) and our measured value is 88 W m^−1^ K^−1^ at room temperature. For pure nickel and the binaries NiCo and NiFe, we used embedded-atom model potentials^32^, whereas for ternaries and quaternaries, we used a simple Lennard–Jones model with parameters related to the chemical species of interest. As alloy complexity increases, more phonon scattering is generally expected. Our calculations for lattice thermal conductivity suggest that the lattice conductivity in NiFe and NiCo is reduced in an equiatomic composition to about 50% of the value corresponding to the linear interpolation of conductivity between the two pure elements. For ternaries and quaternaries, our results suggest that adding more elements does not necessarily further decrease the thermal conductivity. At room temperature, our measured total thermal conductivity for NiCo, NiFe and NiCoFeCr is 69.9, 28.0 and 12.8 W m^−1^ K^−1^, respectively. While the corresponding derived lattice conductivity for NiCo is negligible, the lattice conductivity for NiFe and NiCoFeCr is 7.6 and 4.8 W m^−1^ K^−1^, respectively, comparable to the estimated electrical thermal conductivity of 21.1 and 8.0 W m^−1^ K^−1^, respectively. Also of importance is the fact that the total thermal conductivity at room temperature significantly decreases from 88 W m^−1^ K^−1^ for nickel to 28.0 W m^−1^ K^−1^ for NiFe and 12.8 W m^−1^ K^−1^ for NiCoFeCr.

### Defect dynamics and damage accumulation

The results from electronic structure calculations, electrical and lattice thermal conductivity calculations, as well as the resistivity and thermal conductivity measurements indicate that alloy complexity can have a significant impact on electron–electron scattering, electron–ion coupling and phonon–phonon scattering. The significant modification of the energy dissipation pathways in concentrated solid solution alloys, compared with those in pure nickel, is largely due to the highly localized heat conduction (reduced mean free path) in the electronic subsystem (resulting from the chemical disorder). While the reduction of phonon mean free path is also attributed to the mass and force constant disorder resulting from the chemical disorder, further reduction in the mean free path for phonons may arise from the displacement fluctuation. Less efficient heat conduction in the atomic subsystem is, therefore, expected. Such changes in energy dissipation pathways may affect defect dynamics.

**Figure 3:**
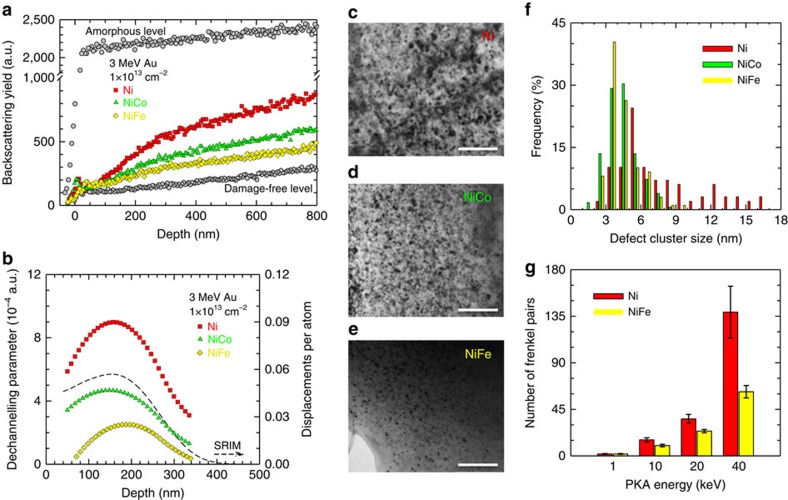
Response to ion energy deposition. (**a**) Rutherford backscattering spectra along (001) direction. Both random and virgin spectra are included to indicate the damage-free level before irradiation and a full amorphous level where no crystallinity exists. (**b**) Ion irradiation-induced damage distribution determined from the ion channelling spectra in **a** and damage profile in nickel predicted using the Kinchon–Pease damage calculation model in the SRIM code. Compared with pure nickel, improved irradiation resistance is observed under 3 MeV Au to fluence of 1 × 10^13^ cm^−2^ in the binary alloys, with NiFe outperforming NiCo. Microstructural characterization was performed where bright-field images were taken from the damage peak region of (**c**) nickel, (**d**) NiCo and (**e**) NiFe samples irradiated using 3 MeV Au ions to a fluence of 2 × 10^13^ cm^−2^, scale bars 40 nm. The size distribution of defect clusters is summarized in (**f**). A comparison of the number of Frenkel pairs produced in nickel and NiFe is shown in (**g**). Ion–solid interactions are stochastic; primary knock-on atoms (PKA) with the same initial energy may result in different number of defects. The corresponding uncertainty is provided as the error bars based on the statistics from the displacement cascade simulations.

The impact of extreme compositional disorder on defect production and radiation response is demonstrated by irradiating three fcc model materials: pure nickel and NiCo and NiFe alloys. Advances in understanding and modelling such ion–solid interactions that mimic both natural and man-made radiation environments are essential to the development of radiation-tolerant materials for nuclear energy, high-energy accelerator and space applications. To predict and control material properties under non-thermal equilibrium environments that are critical to many energy-related technologies, energetic charged particles were utilized to study dynamic response of materials to external energy deposition. Energetic gold (Au) ions were used to perform room-temperature irradiation. High-quality single crystals were used to eliminate the influence or complications of pre-existing conditions, such as grain boundaries and precipitates, which might cause additional reduction of heat conductivity. Heavy Au^2+^ ions at 3 MeV were chosen to effectively produce displacement damage within a few hundred nanometres from the sample surface that is readily measurable using an ion channelling technique. A difference in irradiation response is clearly observed (Fig. 3a); a higher backscattering yield due to higher irradiation damage is evident for the pure metal, indicating that nickel is the most radiation-sensitive crystal among the three crystals. When 50% nickel was substituted with cobalt, the backscattering yield decreases by approximately one-third; when 50% nickel was substituted with iron, the absolute yield is approximately half that obtained for nickel.

To understand the nature of the irradiation-induced damage and confirm the ion channelling results for the defect reduction observed in the binaries, microstructural characterization with TEM was carried out in the samples that were irradiated using 3 MeV Au to a fluence of 2 × 10^13^ cm^−2^ (Fig. 3c–f). The damage profile from the 3 MeV Au irradiation was predicted in displacements per atom (d.p.a.) using the Stopping and Range of Ions in Matter (SRIM) code^34^. For an ion fluence of 1 × 10^13^ cm^−2^, SRIM calculations yield a peak dose of ∼0.057 d.p.a. at a depth of ∼155 nm for the highest Au concentration of ∼6 p.p.m. (parts-per-million, 10^−6^) at∼260 nm. A low-magnification cross-sectional TEM study (not shown) has confirmed that, under this relatively low-fluence irradiation, the damage profile was reasonably predicted by SRIM. A statistical analysis of the defects in nickel, NiCo and NiFe was performed at the damage peak region. TEM images were taken in the damage peak region (at a depth of ∼155 nm from the sample surface) with the foil oriented along (011) direction. Nano-size (∼2–5 nm) vacancy-type stacking fault tetrahedra (SFT) were observed in all irradiated samples as open triangles consisting of {111} planes. Besides these SFT, interstitial-type dislocation loops with average sizes of 7.0, 4.3 and 4.4 nm in the nickel, NiCo and NiFe, respectively, were also detected by high-resolution TEM. Moreover, larger dislocation loops (up to 16 nm in diameter in nickel) were observed by two-beam condition bright-field TEM (not shown). The size distribution of defect clusters, mainly SFT that were visible under the characterization condition (Fig. 3f), skewed towards larger sizes in nickel. Our molecular dynamics results^35^ suggest that the kinetics of formation is considerably slower in NiFe than in pure Ni, indicating that defect migration barriers and extended defect formation energies could be higher in the alloys than pure Ni. As a result, while larger-size clusters were formed in pure Ni, smaller clusters were observed in the alloys, which is consistent with our TEM results (Fig. 3f).

The passage of a MeV ion through a solid material leads to the deposition of substantial kinetic energy in the material and produces a large number of displaced atoms and corresponding vacant lattice sites. Since the energy transferred from an ion to an atomic nucleus in a single collision is often many times greater than the displacement threshold energy of a few tens of eV, the displaced atom can recoil with enough kinetic energy to displace other atoms, creating what is known as an atomic displacement cascade involving many subsequent generations of atomic collisions. Eventually, a large number of atoms within the solid can be set in motion until the energy is dissipated as a rapidly quenched thermal spike within a picosecond timescale. Although many displaced atoms occupy a nearby vacant lattice site (most vacancy–interstitial pairs recombine) during the cascade cooling phase, the net effect of the deposition of ion energy typically results in a number of stable vacancies and atoms in interstitial positions, along with small clusters of these point defects. The survivable defects from cascades may include both vacancy- and interstitial-type dislocation loops, small cavities and SFT. In an ion channelling measurement, interstitials or small amorphous clusters cause direct backscattering of the probing beam that is normally observed as a steep rise in the backscattering yield. As the yield (Fig. 3a) does not show an apparent damage peak around ∼155 nm, the gentle slope indicates that the damage is neither amorphous clusters nor a high concentration of individual interstitials. Vacancies are known to be immobile at room temperature. While the existence of vacancies may affect the directions of the channelled ions, they do not cause direct backscattering. The most detectable ion-induced damage at room temperature may be dislocation loops and stacking faults. Such results agree with our high-resolution TEM observation and literature studies^36^. For the purpose of discussion, the differential dechannelling parameter (Fig. 3b), which is the product of defect density and the dechannelling cross-section (or dechannelling factor)^37^, may be derived, assuming that most of the damage consists of line defects and planar defects, and the dechannelling cross-section does not vary significantly in the ion-modified region. The derived profiles may be viewed as irradiation-induced damage distributions in nickel, NiCo and NiFe. The shape of the derived profiles is in reasonable agreement with the SRIM-predicted profile of displacement damage (Fig. 3b), which indicates that, in the three model crystals, most ion-induced point defects cluster into dislocation loops and SFT. Compared with pure nickel, the much lower values of the dechannelling parameter indicate less damage in the crystalline structure of the binary alloys, in agreement with the TEM characterization (Fig. 3c–e).

To understand how the presence of multiple principal elements in a simple fcc structure may influence damage formation, and to provide insight into the ion irradiation results, molecular dynamics simulations are being used to investigate primary damage formation in displacement cascades in nickel and NiFe. Our molecular dynamics simulations (Fig. 3g) show that stable defect production is higher in nickel than in NiFe by a factor of ∼2, which is qualitatively consistent with the irradiation results (Fig. 3b,c,e,f). The cascade simulations show that only a fraction of the defects that are produced remain as individual vacancies and interstitials. The remaining point defects are found in clusters, with the vacancies primarily in SFT and the interstitials in small dislocation loops of 1/3<111>{111}-type and 1/2<110>{110}-type. This is consistent with our ion channelling measurements and TEM observations. As discussed earlier^35^, our molecular dynamics simulations have shown slower interstitial clustering in NiFe than in pure nickel. This difference may influence the evolution of the cascade-produced clusters and lead to larger clusters in pure nickel and smaller clusters in NiFe. The temperature profile analysis of the molecular dynamics simulation suggests that different thermal diffusion coefficients change the survival times of thermal spikes. Comparing with pure nickel, the lower extent of radiation damage produced in NiFe probably relates to the longer thermal spike lifetime due to less efficient heat conduction, which agrees well with our earlier results from the electronic structure calculations and resistivity measurements (Figs 1 and 2).

### Alloy complexity and damage accumulation

**Figure 4:**
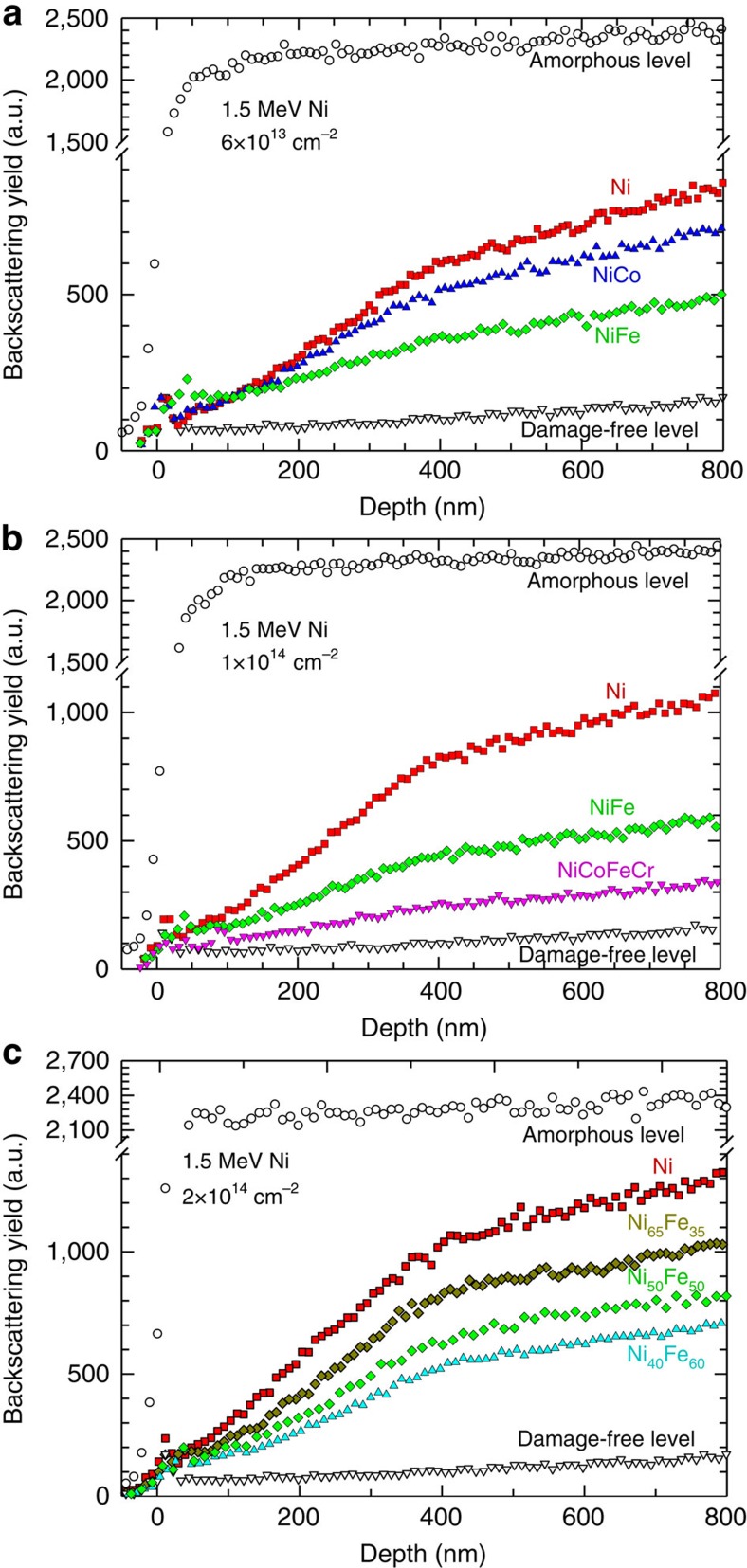
Rutherford backscattering spectra showing different irradiation response in nickel and Ni-based SP-CSAs. (**a**) A change in principal element matters where different damage level is observed after irradiation of 1.5 MeV nickel to a fluence of 6 × 10^13^ cm^−2^ in 50% Ni alloyed with 50% of nickel, cobalt or iron, respectively. (**b**) Alloy complexity matters where in this particular case the more complex system, the more radiation tolerant. (**c**) Composition matters where the higher the iron concentration in the NiFe binary alloys the lower is the damage level observed after 1.5 MeV nickel irradiation to a fluence of 2 × 10^14^ cm^−2^.

If the nature of the specific principal elements is important, what would the impact be if the concentration of the principal elements is changed, or even more challenging, if the alloy of interest moves from a binary to a more complex system? To further validate the hypothesis regarding alloy complexity and to ensure radiation response does not depend on the energetic ions, irradiation experiments using nickel self-ions were carried out on nickel, Ni-based binary and quaternary alloys. Because extreme energy deposition processes from energetic ions can lead to complex defect configurations in alloys with increasing compositional complexity, the relative backscattering yield, instead of the dechannelling parameter, is used for comparison of irradiation responses (Fig. 4). Similar to the irradiation response observed under Au irradiation (Fig. 3a), improved radiation resistance in binaries is confirmed (Fig. 4a) under nickel irradiation. With further irradiation of ion fluences from 6 × 10^13^ to 1 × 10^14^ cm^−2^ and to 2 × 10^14^ cm^−2^ (with peak dose at ∼0.18, 0.3 and 0.6 d.p.a.), an increase in backscattering yield is observed in both nickel and NiFe, respectively (Fig. 4a–c), with NiFe outperforming nickel under all irradiation conditions. Moreover, following irradiation of a high-quality quaternary alloy NiCoFeCr, a further suppression of accumulated damage profile level is observed (Fig. 4b), where the defect density is much lower than that in NiFe and ∼5 times less than in nickel. Furthermore, the concentration of principal elements is also important. An enhanced irradiation resistance is observed (Fig. 4c) with an increase of iron concentration from 35 to 60%, suggesting intrinsic properties of elemental species and local environments are key factors for controlling energy dissipation.

## Discussion

The results shown in Figs 1, 2, 3, 4 indicate that defect production and recombination depend strongly on alloy complexity. With the addition of a principal element (cobalt or iron) or a change from binary to quaternary, much lower defect production and delayed accumulation are observed for the model crystals chosen in this study. The results should, however, not be taken to mean that HEAs are intrinsically more radiation resistant. The important message is that exploiting single-phase concentrated solid solutions of multiple principal elements is an effective approach to modifying intrinsic material transport properties, which can affect equilibrium and non-equilibrium defect dynamics. We propose that control of energy dissipation is a key to affect defect evolution.

Although it has long been observed that specific alloy compositions have higher radiation resistance than their pure metal counterparts, the underlying defect mechanisms remain unclear (Supplementary Note 1). Our results (Figs 1, 2, 3, 4) clearly show that extreme chemical complexity, where the various principal elements substitute randomly for one another in a solid solution with a range of possible compositions, has a substantial impact on energy dissipation processes and thus defect formation and evolution. The effects of solid solution disorder on electronic and atomic properties can effectively modify energy transport under irradiation. This can be either direct, through changes in mean free path, or indirect, through changes in the flow of energy from one subsystem to another via electron–phonon coupling. Clearly, varying the number and the specific atomic species of a SP-CSA has the potential to tune both electron and phonon mean free paths and dissipation mechanisms to affect defect production and recombination. Extreme alloying complexity may also have a profound effect on defect formation energies and migration barriers. Interstitials, vacancies and defect clusters produced during ion energy deposition will be affected by intrinsic chemical disorder and lattice distortion, and additional structural complications (such as pre-existing irradiation-induced damage) will occur as defects evolve, all of which lead to challenging issues worthy of further investigation. Understanding the effects of alloy complexity on energy dissipation and defect evolution will enable us to control energy dissipation in complex alloys. More importantly, can we take advantage of property enhancements resulting from alloying and chemical disorder to devise a structure with an inherent ability to self-recover from irradiation?

In summary, while material properties have steadily progressed from the ancient Bronze Age until now, mankind's knowledge of metallurgy largely focuses on traditional alloys in which the matrix consists of one principal element with one or a few other elements as minor additions. In sharp contrast to traditional alloys, the existence of multiple elemental species in SP-CSAs leads to intrinsic chemical disorder and lattice distortion. Alloy complexity can lead to orders of magnitude reductions in electron mean free paths that greatly reduce the electronic subsystem's effectiveness in energy dissipation. Such localized electron–electron interactions also lead to a prolonged thermal spike and increases in electronic temperature along the ion path and in the vicinity of the collision cascades, and therefore have a substantial impact on defect dynamics. Suppression of damage accumulation is observed with increasing complexity from pure nickel to more complex concentrated solid solution alloys, including NiCo, NiFe and NiCoFeCr. Controlling complexity enables the modification of coupling strengths, control of energy dissipation pathways and rates, and alteration of the defect energetics. As SP-CSAs, including HEAs and the corresponding binary, ternary and quaternary subsystems, possess unique links between energy dissipation through coupled electronic, atomic and magnetic interactions and its impact on atomic processes, they are ideal systems to explore the controlling factors for radiation resistance. Systematic exploration of this novel class of materials can lead to new paradigms for alloy development by providing insights into how chemistry and composition can alter energy dissipation and defect evolution mechanisms that energetically may drive recovery from the damage state. Further exploration can provide a new path forward in the design of radiation resistant alloys, and push material by design to the level of atoms and electrons.

## Methods

### Electronic structure calculations in substitutional alloys

Calculations of the configurationally averaged densities of states and Bloch spectral function along high-symmetry directions for pure nickel and the substitutionally disordered equiatomic NiCo, NiFe and NiCoFeCr alloys were performed using the *ab initio* KKR-CPA electronic structure method^25^,^38^ as implemented in the Hutsepot code. The KKR-CPA method describes the effects of chemical disorder on the electronic states, and delivers the configurationally averaged properties, for example, electronic structure, magnetic structure and transport properties. The calculations were performed for an ideal fcc structure using the experimentally determined lattice parameters: 3.5238, 3.5345, 3.5826 and 3.5715 Å for nickel, NiCo, NiFe and NiCoFeCr, respectively. All calculations were performed using the local spin density approximation for the exchange correlation and the atomic sphere approximation to the crystal potential. Calculations were performed spin polarized, and all of the alloy systems yielded a magnetic ground state solution. An angular momentum cutoff of 3 was used in the solution of the multiple-scattering equations. The KKR-CPA scattering-path matrix was calculated in reciprocal (**k**) space using a 20 × 20 × 20 **k**-point mesh during the density functional theory self-consistency cycle and 50 × 50 × 50 **k**-point mesh for the DOS calculation. The DOS and BSF were calculated at energy of 0.001 Rydberg off into the upper half of the complex plane, which gives rise to the slight broadening of the BSF for pure nickel, as shown in Fig. 1.

### Lattice thermal conductivity calculations

Molecular dynamics simulations were also performed to calculate the lattice thermal conductivity of nickel, NiFe and NiCoFeCr using LAMMPS (Large-scale Atomic/Molecular Massively Parallel Simulator). The simulations were performed using both the embedded-atom model Bonny potential for NiFe^32^ and dimensionless Lennard–Jones potentials for binaries, ternaries and quaternary NiCoFeCr. The sample was a cubic box containing 32,000 atoms with a lattice constant relaxed to its equilibrium value for each case. Simulations were run in the NVT ensemble at zero pressure for equilibrations, starting with a conjugate gradient minimization, followed by a thermal equilibration at temperature *T*=0.2 for 5,000 steps. All simulations used a time step Δ*t*=0.005. Thermal conductivity was determined using the Green–Kubo approach, which requires the velocity auto-correlation function obtained from fluctuations at equilibrium in the NVE ensemble. In Green–Kubo simulations, the thermal conductivity data were obtained after 3 million steps, with ensemble averages done over 10,000 overlapping samples.

### Cascade simulations

Displacement cascades in nickel and NiFe were simulated using the molecular dynamics code LAMMPS (lammps.sandia.gov)^39^. Both nickel and NiFe were described using the appropriate components of the FeNiCr interatomic potential developed by Bonny *et al*.^32^, which includes the universal ZBL repulsive potential at close distance. Cascade energies of 1, 10, 20 and 40 keV were simulated. The simulation box was 40 × 40 × 40 lattice parameters for 1, and 10 keV and 80 × 80 × 80 lattice parameters for 20 and 40 keV. Each simulation was performed in the NVE (constant mass, volume and temperature) ensemble for 20 to 35 ps. The atom type, nickel or iron, on the fcc lattice sites in NiFe was randomly selected. The system was equilibrated at 300 K and zero pressure before the simulation. Initial velocities were set using different random seeds. The selected primary knock-on atoms that initiated the cascades were given velocity along the [135] direction. A Langevin bath was applied at the borders of the box, extending four lattice parameters inside it. A variable time step was used to limit displacements to a maximum of 0.014 Å per step. Defect identification was performed using Wigner–Seitz cell analysis. The structure of defects was assessed identifying atoms lying more than 1 Å from a lattice site. The software package OVITO^40^ was used for visualization. For thermal analysis, a mesh was built in which temperature was computed using Gaussian kernel averaging of atomic velocities. This mesh was then used to compute concentric spherical temperature profiles around the cell with the highest initial temperature.

### Crystal growth

Ni-based single-phase concentrated solid solution alloys were prepared using high-purity elemental metals by arc melting and drop casting into cylindrical copper moulds with diameters of ∼12 mm. The phase stability and the formation of single phases were carefully examined and confirmed using X-ray diffraction and microstructure characterization techniques^41^. Single crystals of pure nickel and equiatomic NiFe, NiCo and NiCoCrFe, as well as non-equiatomic Ni_0.65_Fe_0.35_ and Ni_0.40_Fe_0.60_, were directionally solidified to produce single crystals in an optical floating zone furnace from polycrystalline drop-cast rods under an Ar atmosphere^42^. During single-crystal growth, the diameter of the molten zone was carefully controlled to produce a neck that prevents the more slowly growing grains from propagating^43^,^44^. The quality and orientation of all crystals were inspected using backscatter Laue diffraction, reoriented and cut normal to the [100] directions by electro-discharge machining. For Rutherford backscattering spectrometry measurements along the (001) channelling direction, all crystals were carefully polished to produce damage-free surfaces.

### Ion energy deposition

Energy was deposited in the model systems by irradiation with 3 MeV Au^2+^ and 1.5 MeV Ni^+^ ions. The irradiations were performed at a few degrees off the surface normal to avoid channelling implantation. The irradiation-induced damage profile in terms of d.p.a. was calculated using the SRIM code^34^ with the Kinchon–Pease damage calculation model, measured sample densities of 8.908, 8.8484, 8.2326 and 8.1435, g cm^−3^ for nickel, NiCo, NiFe and NiCoFeCr, respectively, and an assumed threshold displacement energy of 40 eV for all elements^45^. It is worth noting that the d.p.a. values obtained using the Kinchon–Pease model are consistent with the values calculated using standard reference models, while the results of the full cascade damage model in SRIM are roughly a factor of two higher^46^.

### Ion beam analysis

Irradiation-induced damage in crystalline samples was quantified utilizing a channelling Rutherford backscattering spectrometry technique. A parallel 3.5 MeV He beam was employed in the measurement that was well aligned in <001> crystal direction, nearly perpendicular to the sample surface. If a crystal contains displaced lattice atoms, or interstitial- or vacancy-type clusters, the nominal open channels from ordered lattice atoms in a prefect crystal will be distorted and the backscatter yield will increase, resulting in both direct backscattering and enhanced dechannelling of the probing ions. Following the ion energy deposition by either energetic Au ions or Ni ions, *in situ* channelling measurements were subsequently carried out with a Si detector located at a scattering angle of 155° relative to the incoming He beam.

### Microstructural characterization

Sample preparation for transmission electron microscopy analysis was done using a focused ion beam (FIB) in FEI Helios 650 Nanolab. A flash polishing technique was developed to remove FIB-induced damage that may easily overlap with the damage from the primary ion irradiation. The standard ‘lift-out' procedure for the cross-sectional TEM sample preparation was first conducted using a gallium FIB beam probe with a current of 2.5 nA at 30 keV. The current was gradually reduced to 30 pA to minimize FIB beam damage while thinning the lamella to ∼100 nm. Final thinning and cleaning were performed at 5 keV with an incidence angle of ±7° for 5 min. The flash polishing was conducted using an electro-polishing apparatus, with an electrolytic solution of 4% perchloric acid in 96% ethanol. An electric potential of 6–14 V was applied to the FIB-polished specimen in a temperature range from −30 to −50 °C for 0.05 to 0.2 s. Bright-field imaging of the same samples was taken in a JEM 3011TEM operating at 300 kV with an exposure time of 2 s.

## Additional information

**How to cite this article:** Zhang, Y. *et al*. Influence of chemical disorder on energy dissipation and defect evolution in concentrated solid solution alloys. *Nat. Commun.* 6:8736 doi: 10.1038/ncomms9736 (2015).

## Supplementary Material

Supplementary Information Supplementary Note 1 and Supplementary References
