# Supplementary material for: Influence of chemical disorder on energy dissipation and defect evolution in concentrated solid solution alloys
Source: Nat Commun. 2015 Oct 28;6:8736. doi: 10.1038/ncomms9736 (PMC4640100; doi:10.1038/ncomms9736)
Supplement: Supplementary Information — Supplementary Note 1 and Supplementary References [file ncomms9736-s1.pdf]

<u>**Supplementary Note 1**</u>

Overview of Alloying Effects

In the long history of alloy development, numerous experimental observations have shown that certain levels of substitutional additions in binary and ternary alloys can significantly affect radiation performance. For example, the Ni–Cu system displays a remarkable resistance to void formation under irradiation (*1,2*). In pure nickel, fewer voids form under ion irradiation with Cu ions than with Ni ions, indicating possible chemical effects of substituting Cu into the nickel lattice. In the Ni–Al binary alloy system (*3*), radiation-induced density reduction depends profoundly on both Al concentration and irradiation temperature (400 – 650°C), where the progressive addition of Al (1.1–13.3 wt%) decreases the saturation swelling at lower temperatures. At higher temperatures, however, swelling tends to increase again at higher Al concentrations. Although the importance of Al content is clearly demonstrated, the underlying mechanisms of energy dissipation and temperature-dependent defect migration are not known. Commercial high–strength Ni–Cr systems have been developed in the 1950s–1960s for jet engine turbine blades. Properties of some Ni–Cr alloys (with and without 1% Mo) have been studied to determine their suitability for use as structural materials for nuclear reactors (*4,5*). While the absence of gas–vacancy cavities in these alloys indicates a low concentration of vacancies and low mobility of transmutant He, the low dislocation density suggests a low concentration of surviving interstitial atoms. Moreover, microstructural characterization (*6*) has also shown that addition of Cr in nickel leads to suppression of defect production under low energy self-ion irradiation. This low quasi–stationary concentration of primary point defects can be viewed as a result of either reduced defect production or enhanced recombination, which may be an intrinsic feature associated with the alloy composition and local chemistry. The atomic size and chemical effects of solute atoms (Si, Mn, Co, Cu, Nb, and Pd with concentration up to 5%) have been studied in He–irradiated nickel and Ni-based binary alloys at 500°C (*7*), and the swelling results show that the number density of bubbles and their mean size increased with the solute size mismatch. Such observations imply that both the irradiation–induced point defects and the mobility of He are influenced by elemental species, lattice distortion, and chemical disorder. The strong influence of solute size on irradiation response has also been observed in the V–Fe binary and V–Fe–Ti ternary systems (*8*). The void swelling of pure vanadium is low after irradiations up to ~11 dpa. Iron additions (5%) to vanadium lead to dramatic increases in swelling, up to 30%. In contrast, adding low levels of Ti to the V–5Fe alloy leads to suppression of swelling. A similar result has been reported recently in V–Ti and V–Fe alloys under He irradiation (*9*). The arguments, based on the impact of undersize (Fe replacing V) and oversize (Ti replacing V) solutes for the observed radiation sensitivity, clearly demonstrate the significant impact of unique site-to-site lattice distortions and local disordered chemical environments due to alloy additions. With increasing complexity of elemental components and chemical disorder, defect production and damage evolution in more complex solid solution alloys show new features. In face-centered cubic alloys, point defect dynamics in neutron–irradiated nickel, Fe–15Cr–16Ni, and Ti modified SUS316SS have been recently investigated (*10*). Although more interstitial–type dislocation loops are observed at lower temperatures in nickel than in Fe–15Cr–16Ni and the modified SUS316, higher swelling is observed in nickel than in Fe–15Cr–16Ni, and no voids are observed in the modified SUS316. The significant difference in defect formation and damage evolution between the three-component Fe–Cr–Ni alloy and the more complex SUS316 is attributed to the additional Ti and low concentration of C. These results demonstrate that chemical complexity, even due to the addition of low solute concentrations, can have a profound effect on fundamental processes controlling defect production and damage evolution. In this long history of alloy development, significant property enhancements postulate underlying mechanisms by which local structural distortion and chemical disorder could affect energy dissipation and thus control defect dynamics. Understanding how material properties can be tailored by alloy complexity and their influence on defect dynamics may pave the way for new design principles of radiation–tolerant structural alloys for advanced energy systems.

Supplementary References

1. Wang, L. M., Dodd, R. A. & Kulcinski, G. L. Gas effects on void formation in 14 MeV nickel ion irradiated pure nickel. *J. Nucl. Mater.* **141-143**, 713-717 (1986).
2. Wang, L. M., Dodd, R. A. & Kulcinski, G. L. Radiation damage and copper distribution in 14 MeV copper-ion-implanted nickel — TEM and AEM analyses in cross-section. *Ultramicroscopy* **29**, 284-290 (1989).
3. Mitchell, M. A. & Garner, F. A. Neutron-induced swelling of binary Ni-Al alloys. *J. Nucl. Mater.* **187**, 103-108 (1992).
4. Skylaris, C. K., Haynes, P. D., Mostofi, A. A. & Payne, M. C. Introducing ONETEP: linear-scaling density functional simulations on parallel computers. *J. Chem. Phys.* **122**, 84119 (2005).
5. Kolotushkin, V. P. & Votinov, S. N. Structural factor and effects of low-temperature radiation damage in structural materials. *Met. Sci. Heat Treat.* **48**, 452-458 (2006).
6. Robinson, T. M. & Jenkins, M. L. Heavy-ion irradiation of nickel and nickel alloy. *Philos. Mag. A* **43,** 999 (1981)
7. Wakai, E. *et al.* Effect of solute atoms on swelling in Ni alloys and pure Ni under He+ ion irradiation. *J. Nucl. Mater.* **307-311**, 367-373 (2002).
8. Fukumoto, K., Kimura, A. & Matsui, H. Swelling behavior of V–Fe binary and V–Fe–Ti ternary alloys. *J. Nucl. Mater.* **258-263**, 1431-1436 (1998).
9. Chernov, I. I. *et al.* Mechanisms of helium porosity formation in vanadium alloys as a function of the chemical composition. *Atom. Energy* **109**, 176-183 (2011).
10. Horiki, M., Yoshiie, T., Sato, K. & Xu, Q. Point defect processes in neutron irradiated Ni, Fe–15Cr–16Ni and Ti-added modified SUS316SS. *Philos. Mag.* **93**, 1701-1714 (2013).
